# Multitarget Antiproliferative Activity of *Pituranthos scoparius*: An Integrated Phytochemical, Biological and Computational Insights into Key Oncogenic Pathways

**DOI:** 10.3390/molecules31152561

**Published:** 2026-07-23

**Authors:** Sarra Chabane, Amel Boudjelal, Luana Pulvirenti, Aslı Yıldırım Kocaman, Ibrahim Demirtas, İlyas Yıldız, Fatih Gül, Süleyman Muhammed Çelik, Amrane Abdeltif

**Affiliations:** 1 Department of Veterinary, Faculty of Sciences, University of M’sila, M’sila 28000, Algeria; sarra.chabane@univ-msila.dz; 2 Laboratory of Biology: Applications in Health and Environment, University of M’sila, M’sila 28000, Algeria; 3 Department of Microbiology and Biochemistry, Faculty of Sciences, University of M’sila, M’sila 28000, Algeria; 4 Institute of Biomolecular Chemistry (ICB), National Research Council of Italy, Via Paolo Gaifami 18, 95126 Catania, Italy; 5 Department of Health Services, Vocational School of Higher Education for Healthcare Services, Igdir University, 76000 Igdir, Türkiye; asli.yildirim60@gmail.com; 6 Department of Pharmaceutical Chemistry, Faculty of Pharmacy, Ondokuz Mayıs University, 55270 Samsun, Türkiye; ibdemirtas@gmail.com; 7 Ecole Nationale Supérieure de Chimie de Rennes, CNRS, L’Institut des Sciences Chimiques de Rennes (ISCR–UMR 6226), University of Rennes, 35000 Rennes, France; lysyldz60@gmail.com; 8 Department of Molecular Biology and Genetics, Tokat Gaziosmanpasa University, 60000 Tokat, Türkiye; fatih.gul@igdir.edu.tr; 9 Research Laboratories and Research Application Center (ALUM), Igdir University, 76000 Igdir, Türkiye; suleymancelik10@gmail.com; 10 Department of Medical Services and Techniques, School of Health Services, Igdir University, 76000 Igdir, Türkiye; abdeltif.amrane@univ-rennes.fr

**Keywords:** antiproliferative activity, multitarget, natural products, molecular docking, PI3Kα/mTOR pathway

## Abstract

***Background***: *Pituranthos scoparius* (*Apiaceae*), commonly known in Algeria as “Kozah”, is a medicinal plant traditionally used for various therapeutic purposes. However, its potential as a source of antiproliferative agents and its underlying molecular mechanisms remain poorly characterized. ***Purpose***: This study aimed to investigate the antiproliferative potential of *P. scoparius* through an integrated strategy combining phytochemical profiling, in vitro evaluation, and computational approaches, with particular emphasis on its activity against key cancer-related pathways. ***Study Design***: An integrated experimental–computational study was performed to explore the multitarget antiproliferative profile of *P. scoparius* extracts. ***Methods***: Phytochemical characterization was carried out using LC-MS/MS and GC-MS/MS to identify the major bioactive constituents. Antiproliferative activity was assessed in vitro against human colorectal (HT-29) and hepatocellular carcinoma (HepG2) cell lines using the MTT assay. Molecular docking studies were conducted on selected major metabolites against relevant oncogenic targets, including PI3Kα, mTOR, COX-2, and BCL-2, to investigate potential mechanisms of action. Machine learning approaches were further employed to support the prediction of multitarget antiproliferative activity. ***Results***: Chlorogenic acid and *trans*-ferulic acid were identified as the predominant phenolic compounds, while α-pinene was the major constituent of the essential oil. Both preparations exhibited significant dose- and time-dependent antiproliferative effects, with the essential oil showing enhanced cytotoxicity (IC_50_ up to 35.4 µg/mL). In silico analyses revealed strong binding affinities of key metabolites toward critical oncogenic proteins, particularly within the PI3Kα/mTOR signaling pathway. Machine learning predictions further supported a multitarget antiproliferative profile. ***Conclusions***: *P. scoparius* represents a promising source of natural compounds with multitarget antiproliferative potential. The combined experimental and computational findings provide mechanistic insights into its activity and support its relevance within the context of natural product-based cancer therapy, highlighting its potential for further preclinical development.

## 1. Introduction

Cancer is a complex disease characterized by uncontrolled cell proliferation, genomic instability, and metastatic potential, and it represents a major global public health challenge. According to estimates reported by the International Agency for Research on Cancer, approximately 20 million new cancer cases and 9.7 million cancer-related deaths occurred worldwide in 2022. Future projections suggest that global cancer incidence could exceed 35 million new cases annually by 2050, representing an increase of nearly 77% compared with current levels, according to the GLOBOCAN [1]. Among the most prevalent malignancies, colorectal cancer accounts for approximately 10–15% of all cancers worldwide and represents the most common malignancy of the gastrointestinal tract [2], whereas liver cancer remains the third leading cause of cancer-related mortality globally [3]. In recent years, herbal medicine has attracted increasing scientific interest as a valuable source of structurally diverse bioactive metabolites with potential antiproliferative properties. Medicinal plants are increasingly recognized as important reservoirs of bioactive compounds with therapeutic potential in oncology [4]. Recent evidence further highlights the growing relevance of herbal medicines as sources of anticancer agents and chemopreventive compounds [5]. In particular, multitarget approaches are increasingly recognized as a promising strategy in cancer therapy, as they may overcome resistance mechanisms associated with single-target drugs and modulate complex oncogenic networks. Natural products can simultaneously interact with multiple molecular targets involved in proliferation, apoptosis, inflammation, and metastasis, supporting their potential application in multitarget anticancer therapy [6]. The integration of advanced phytochemical profiling techniques with biological assays and computational approaches has therefore emerged as an effective strategy to correlate chemical composition with pharmacological activity [7,8]. However, despite increasing interest in *P. scoparius*, studies integrating comprehensive phytochemical profiling with mechanistic interpretation of its antiproliferative activity and underlying molecular mechanisms remain poorly understood. In particular, the contribution of individual phytoconstituents within a phytocomplex framework and their potential multitarget interactions are still poorly understood. Medicinal plants represent an important reservoir of bioactive compounds with potential therapeutic applications, particularly in oncology. Among these species, *P. scoparius* (*Apiaceae*) is an endemic plant that grows spontaneously in arid and semi-arid regions of North Africa. In Algeria, it is widely distributed in rocky pastures and high plateau regions of the Sahara [9]. Traditionally, this species has been used for its antimicrobial, antioxidant, anti-inflammatory, and digestive properties, and is also employed in the management of asthma, rheumatism, spasms, diabetes, urinary infections, and envenomation. The traditional use of *P. scoparius* for inflammatory and metabolic disorders provides a strong ethnopharmacological rationale for investigating its potential antiproliferative properties, considering the well-established link between chronic inflammation and tumor progression. Although several studies have reported some biological activities of this species, comprehensive phytochemical characterization combined with mechanistic evaluation of its antiproliferative potential remains limited. In this context, molecular modeling approaches have gained increasing attention as complementary tools to experimental studies. Structure-based molecular docking enables the prediction of interactions between natural compounds and their derivatives with key biological targets, thereby providing valuable mechanistic insights into their pharmacological effects [8,10]. Therefore, the present study aimed to (i) characterize the phytochemical composition of the methanolic extract and essential oil of *P. scoparius* using LC-MS and GC-MS analyses, (ii) evaluate their antiproliferative activity against colorectal cancer (HT-29) and hepatocellular carcinoma (HepG2) cell lines, and (iii) investigate potential molecular targets through an integrated molecular docking approach in order to provide a mechanistic interpretation of the observed biological effects. The findings of this research could contribute to the development of new plant-based therapeutic agents for cancer treatment.

## 2. Results and Discussion

### 2.1. Phytochemical Characterization

#### 2.1.1. LC–MS/MS Analysis of the Methanolic Extract

**Figure 1:**
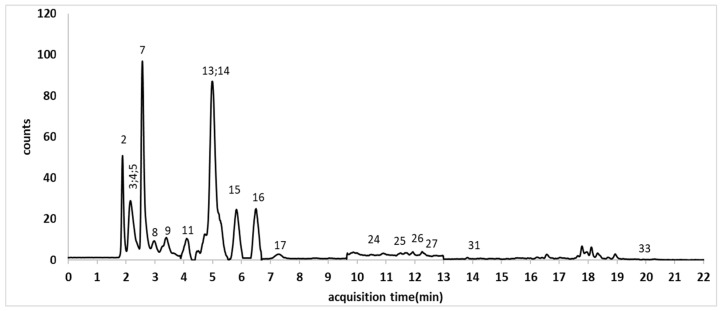
Representative LC–MS/MS chromatogram of the methanolic extract of *Pituranthos scoparius*. Only the major and clearly distinguishable peaks are labelled in the chromatogram for clarity. The complete peak assignment, tentative identification, retention times, and quantitative data for all detected compounds are reported in Table 1.

The results of LC–MS/MS analysis of the *Pituranthos scoparius* methanolic extract and the corresponding chromatogram are presented in Table 1 and Table 2 and Figure 1, respectively. Quantitative analysis revealed chlorogenic acid (191.41 µg/g extract) as the predominant constituent, followed by *trans*-ferulic acid (96.2 µg/g extract) and vanillic acid (39.28 µg/g extract). Moderate concentrations were observed for gentisic acid (18.93 µg/g extract), sinapic acid (16.67 µg/g extract), salicylic acid (12.07 µg/g extract), and *p*-coumaric acid (9.05 µg/g extract), whereas flavonoids such as kaempferol (8.79 µg/g extract) and rutin (7.73 µg/g extract) were detected at lower levels. Compounds not detected under the applied analytical conditions were excluded from quantitative interpretation, and only analytes producing measurable signals above the established detection threshold were considered.

Total phenolic acids identified in the methanolic extract of *P. scoparius* include gallic acid, chlorogenic acid, gentisic acid, caffeic acid, syringic acid, vanillic acid, *p*-coumaric acid, sinapic acid, *trans*-ferulic acid, salicylic acid, *o*-coumaric acid, and protocatechuic acid. Hydroxycinnamic acids, as a subclass, comprise chlorogenic acid, caffeic acid, *p*-coumaric acid, sinapic acid, *trans*-ferulic acid, *trans*-cinnamic acid, and *o*-coumaric acid. The flavonoid fraction includes epigallocatechin, catechin, rutin, isoquercitrin, taxifolin, baicalin, quercetin, naringenin, kaempferol, morin, 7-hydroxyflavone, chrysin, luteolin, biochanin A, and 5-hydroxyflavone. Phenolic aldehydes (hydroxybenzaldehyde and vanillin) and the phenylpropanoid *trans*-cinnamic acid were also detected.

Total polyphenols were calculated as the sum of all quantified phenolic acids, flavonoids, phenolic aldehydes, and phenylpropanoids, excluding diosgenin (2.7 ± 0.1 µg/g), a steroidal saponin detected in the extract. While diosgenin is part of the overall phytochemical profile, it does not contribute to the polyphenol total, which amounted to 422.6 ± 8.9 µg/g extract.

Quantitative analysis showed that the extract is particularly rich in phenolic acids (401.0 ± 10.8 µg/g), mainly represented by hydroxycinnamic acid derivatives (321.7 µg/g), likely the main contributors to its antioxidant and biological activities. Among the identified compounds, chlorogenic acid (191.4 ± 4.8 µg/g) was the predominant metabolite, followed by *trans*-ferulic acid (96.2 ± 2.5 µg/g) and vanillic acid (39.3 ± 1.2 µg/g). Moderate amounts of gentisic acid (18.9 ± 0.8 µg/g), sinapic acid (16.7 ± 0.6 µg/g), salicylic acid (12.0 ± 0.4 µg/g), and *p*-coumaric acid (9.1 ± 0.4 µg/g) were also detected. Flavonoids were present at lower levels (21.6 ± 0.8 µg/g), with kaempferol (8.8 ± 0.3 µg/g) and rutin (7.7 ± 0.3 µg/g) as the major constituents.

Chlorogenic acid was identified as the predominant phenolic constituent in the methanolic extract. Previous studies have reported that chlorogenic acid may influence cellular pathways associated with proliferation, apoptosis, and oxidative stress. Considering its abundance and its predicted interactions with selected molecular targets in the docking analysis, chlorogenic acid may contribute to the antiproliferative activity observed in the present study. However, the current findings do not provide direct evidence that chlorogenic acid alone is responsible for the observed biological effects.

*Trans*-ferulic acid was the second most abundant phenolic compound detected in the extract. This compound has been reported to modulate cellular redox balance and signaling pathways involved in cell survival and proliferation. Although molecular docking suggested potential interactions with selected cancer-related targets, the present results do not allow a direct causal relationship between *trans*-ferulic acid and the observed antiproliferative activity to be established. Vanillic acid was detected at a relatively high concentration among the identified phenolic compounds. Previous reports have suggested that this metabolite may influence cellular responses associated with oxidative stress and cell viability. Nevertheless, no direct experimental evidence was obtained in the present study to demonstrate that vanillic acid individually contributes to the antiproliferative effects observed in HepG2 and HT-29 cells. The observed antiproliferative activity of *P. scoparius* is likely associated with the synergistic effects of multiple phytochemicals present in the extract. Accordingly, the major identified constituents may contribute to this activity, although their individual roles have not yet been experimentally confirmed. Overall, the antiproliferative activity observed for *P. scoparius* is likely associated with the combined and potentially synergistic action of multiple phytochemicals rather than with a single identified constituent.

Overall, these results confirm that the methanolic extract of *P. scoparius* is a rich source of phenolic compounds with potential health-promoting effects.

Table 2 presents the multiple reaction monitoring (MRM) parameters and calibration data used for the quantification of the 33 targeted phenolic and flavonoid compounds. For each analyte, precursor and product ions, dwell time (ms), fragmentor voltage (V), collision energy (CE, V), ionization polarity (positive or negative), and the correlation coefficient (R^2^) of the calibration curve are reported. Calibration equations are also provided, demonstrating the linearity and accuracy of the quantification (Table 2). These parameters ensured sensitive and selective detection of each compound under the applied analytical conditions.

Table 2 presents the multiple reaction monitoring (MRM) parameters and calibration data employed for the quantification of 33 phenolic and flavonoid compounds by LC–MS/MS. For each analyte, the table reports the precursor and product ions, dwell time, fragmentor voltage, collision energy, ionization polarity, and the correlation coefficient (R^2^) of the calibration curve. These carefully optimized conditions enabled sensitive and selective detection, ensuring accurate and reproducible quantification of both phenolic acids and flavonoids in the methanolic extract of *P. scoparius.* The chromatographic profile (Figure 1) indicates that the major phenolic constituents elute within the 2–8 min retention time window under the applied gradient system, consistent with the quantitative data presented in Table 1 and Table 2.

The chromatographic profile (Figure 1) shows that the major phenolic constituents elute within the 2–8 min retention time window under the applied gradient system, consistent with the quantitative data reported in Table 1 and Table 2.

The predominance of hydroxycinnamic acids and related phenolic derivatives classifies the extract as a phenolic-rich phytocomplex, potentially contributing to the observed antiproliferative activity.

Previous studies on polar extracts of *P. scoparius* from the Ain Diss region in Algeria reported high contents of 5-*O*-caffeoylquinic acid and 5-*O*-feruloylquinic acid [11]. The high abundance of chlorogenic acid in the present extract is in agreement with these reports, although quantitative differences may arise from geographical origin, harvesting period, or extraction conditions. Chlorogenic acid, the major component, has been reported to exert antioxidant, anti-inflammatory, and antiproliferative activities, partly through modulation of oxidative stress and cell-signaling pathways involved in proliferation and apoptosis [12]. Similarly, *trans*-ferulic acid can regulate inflammatory mediators, maintain redox homeostasis, and interfere with pathways associated with tumor cell growth and survival [13]. Vanillic acid, also present at significant levels, has been associated with antioxidant and antiproliferative effects in various experimental models [14]. Taken together, the predominance of these hydroxycinnamic acid derivatives suggests that the observed biological activity may result from a combined phenolic-driven modulation of redox balance and cell signaling, rather than from the action of a single compound.

#### 2.1.2. GC–MS/MS Analysis of the Essential Oil

**Figure 2:**
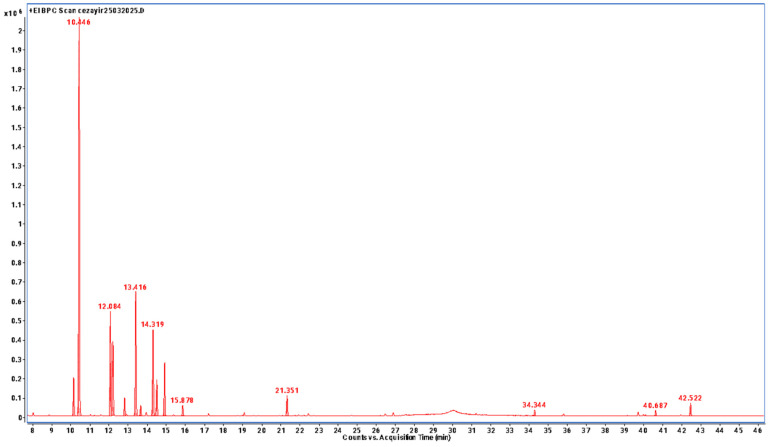
GC–MS total ion chromatogram (TIC) of the essential oil obtained from *P. scoparius*. Major identified peaks correspond to α-thujene (10.15 min), α-pinene (10.45 min), sabinene (12.08 min), β-pinene (12.21 min), β-myrcene (12.83 min), α-phellandrene (13.41 min), 3-carene (13.67 min), p-cymene (14.32 min), β-terpinene (14.52 min), β-ocimene (14.93 min), γ-terpinene (15.88 min), and 4-terpineol (21.35 min), *trans*-ligustilide (42.52 min). Minor constituents (<1%) as well as full peak identification, retention times, relative percentages and other identified compounds are listed in Table 3.

The chemical composition of the essential oil obtained from the aerial parts of *Pituranthos scoparius* was characterized by GC–MS/MS analysis, leading to the identification of 22 constituents, as summarized in Table 3 and illustrated in Figure 2. The volatile profile was dominated by monoterpene hydrocarbons, which constituted the major chemical class of the essential oil. Among them, α-pinene (38.90%), α-phellandrene (12.24%), and sabinene (10.22%) were the predominant components, followed by p-cymene (8.80%) and β-pinene (7.20%). Other constituents were detected in lower amounts, each representing less than 5% of the total composition.

Previous studies have reported qualitative and quantitative variability in the essential oil composition of *P. scoparius*, depending on geographical origin [15]. Samples collected from Tunisia and various Algerian regions, including M’sila, Batna, Biskra, and Souk Ahras, were characterized by relatively high levels of α-pinene (8.3–35.8%) and sabinene (14.8–34.4%). The predominance of α-pinene observed in the present study (38.90%) falls within the upper range of previously described chemotypes, supporting the classification of the analyzed sample as an α-pinene–rich profile. Monoterpenes, the dominant class of compounds in this essential oil, are widely recognized for their diverse biological activities, including antimicrobial, antioxidant, anti-inflammatory, and antiproliferative effects [16]. Among them, α-pinene has been reported to exhibit notable antimicrobial, anti-inflammatory, antioxidant, and antiproliferative properties [17]. In particular, its antiproliferative activity has been associated with the modulation of oxidative stress and apoptosis-related pathways, suggesting a possible contribution to the cytotoxic effects observed in the present study.

### 2.2. Antiproliferative Activity of the Methanolic Extract and Essential Oil

#### 2.2.1. IC_50_ Determination

The antiproliferative effects of the methanolic extract (ME) and essential oil (EO) of *Pituranthos scoparius* were evaluated against HT-29 and HepG2 cell lines using the MTT assay, and the IC_50_ values were determined after 24 and 48 h of treatment (Table 4). Cell viability percentages were calculated, and the effects of each sample at different concentrations were compared with those of the positive control. All results are expressed as mean ± SD from independent experiments and were normalized to the negative control. Both preparations exhibited time-dependent cytotoxic effects, with higher antiproliferative activity observed after 48 h compared to 24 h.

The IC_50_ values presented in Table 4 indicate a clear variation in antiproliferative activity depending on the tested preparation, cell line, and exposure time. Overall, the essential oil exhibited stronger cytotoxic activity than the methanolic extract, particularly after 48 h of treatment. In HepG2 cells, the essential oil showed moderate activity at 24 h (IC_50_ = 110.7 ± 9.09 µg/mL), which markedly increased after 48 h (IC_50_ = 54.89 ± 11.24 µg/mL), indicating a time-dependent enhancement of cytotoxicity. In contrast, the methanolic extract showed detectable activity only at 48 h (IC_50_ = 95.16 ± 10.86 µg/mL), suggesting weaker and slower antiproliferative effects on this cell line. A similar trend was observed in HT-29 cells, where the essential oil demonstrated stronger activity compared with the methanolic extract. The IC_50_ values decreased from 80.94 ± 10.55 µg/mL at 24 h to 35.4 ± 4.57 µg/mL at 48 h, highlighting a pronounced time-dependent effect. No measurable IC_50_ values were obtained for the methanolic extract in HT-29 cells within the tested concentration range, indicating limited activity under the experimental conditions. As expected, the positive control 5-fluorouracil (5-FU) displayed significantly stronger cytotoxicity than both plant preparations, with IC_50_ values decreasing from 62.77 ± 6.80 to 9.89 ± 2.48 µg/mL in HepG2 cells and from 55.65 ± 3.19 to 17.94 ± 2.33 µg/mL in HT-29 cells between 24 and 48 h. This confirms the sensitivity of the assay and provides a reference for the relative activity of the tested extracts. Taken together, these results suggest that the essential oil possesses greater antiproliferative potential than the methanolic extract, particularly against HT-29 cells after prolonged exposure, while the methanolic extract exhibits a more limited and selective activity toward HepG2 cells.

#### 2.2.2. Dose- and Time-Dependent Effects

**Figure 3:**
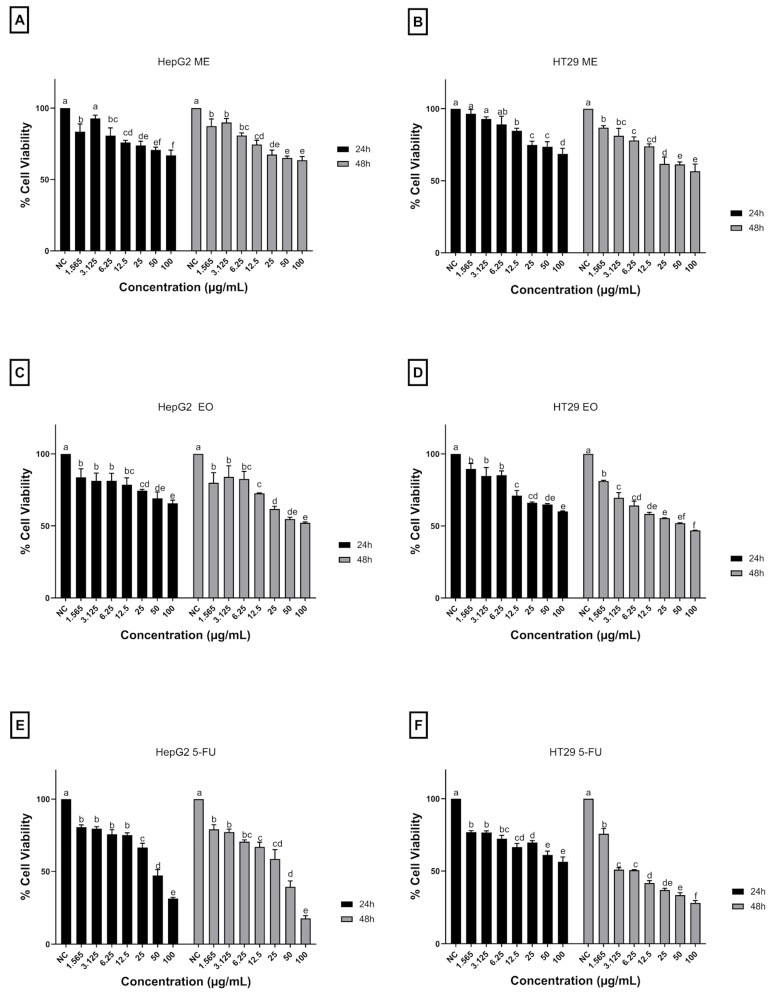
Effects of the methanolic extract (ME), essential oil (EO), and 5-fluorouracil (5-FU) on HepG2 and HT29 cell viability after 24 and 48 h of treatment. (**A**) HepG2 cells treated with the methanolic extract (ME); (**B**) HT29 cells treated with the methanolic extract (ME); (**C**) HepG2 cells treated with the essential oil (EO); (**D**) HT29 cells treated with the essential oil (EO); (**E**) HepG2 cells treated with 5-fluorouracil (5-FU); (**F**) HT29 cells treated with 5-fluorouracil (5-FU). Cell viability was assessed by the MTT assay following exposure to increasing concentrations (1.565–100 μg/mL) for 24 and 48 h. Data are presented as mean ± SD (*n* = 3) and normalized to the negative control (NC). Different letters indicate significant differences among concentrations within the same exposure period (*p* < 0.05).

As illustrated in Figure 3, both the methanolic extract (ME) and essential oil (EO) induced concentration- and time-dependent decreases in cell viability in HepG2 and HT29 cells. In general, a more pronounced cytotoxic effect was observed after 48 h of exposure compared with 24 h, indicating a clear time-dependent response. Across all treatments, cell viability progressively decreased with increasing concentrations (1.565–100 μg/mL), supporting a dose-dependent antiproliferative activity. In HepG2 cells, ME treatment resulted in a gradual reduction in cell viability at both exposure periods. Two-way ANOVA revealed significant effects of concentration (F(7,30) = 95.35, *p* < 0.0001) and exposure time (F(1,30) = 4.88, *p* = 0.0349), whereas the concentration × time interaction was not significant (F(7,30) = 1.81, *p* = 0.1213). The methanolic extract exhibited moderate but selective activity toward HepG2 cells, reaching an IC_50_ value of 95.16 ± 10.86 μg/mL after 48 h of treatment. EO treatment significantly reduced HepG2 cell viability, with significant effects of concentration (F(7,32) = 60.62, *p* < 0.0001), exposure time (F(1,32) = 23.10, *p* < 0.0001), and concentration × time interaction (F(7,32) = 4.23, *p* = 0.0021). In agreement with these findings, EO demonstrated enhanced potency after prolonged exposure, yielding an IC_50_ value of 54.89 ± 11.24 μg/mL at 48 h. In HT29 cells, both ME and EO induced marked reductions in cell viability in a concentration-dependent manner. ME treatment significantly affected cell viability through concentration (F(7,29) = 89.99, *p* < 0.0001), exposure time (F(1,29) = 100.67, *p* < 0.0001), and their interaction (F(7,29) = 2.39, *p* = 0.0467). EO treatment also produced significant effects of concentration (F(7,31) = 291.36, *p* < 0.0001), exposure time (F(1,31) = 322.24, *p* < 0.0001), and concentration × time interaction (F(7,31) = 10.76, *p* < 0.0001). Notably, EO exhibited the strongest activity against HT29 cells, with an IC_50_ value of 35.4 ± 4.57 μg/mL after 48 h of treatment. The positive control 5-fluorouracil (5-FU) displayed the highest antiproliferative activity in both cell lines. In HepG2 cells, significant effects of concentration (F(7,27) = 438.73, *p* < 0.0001), exposure time (F(1,27) = 47.70, *p* < 0.0001), and concentration × time interaction (F(7,27) = 3.85, *p* = 0.0050) were observed. Similarly, in HT29 cells, 5-FU treatment produced highly significant effects of concentration (F(7,32) = 565.12, *p* < 0.0001), exposure time (F(1,32) = 1350.54, *p* < 0.0001), and concentration × time interaction (F(7,32) = 64.91, *p* < 0.0001). Overall, the results demonstrate that both ME and EO possess notable antiproliferative activity against HepG2 and HT29 cancer cells, although their effects were generally less pronounced than those of 5-FU. Statistically significant differences among treatment concentrations were observed, particularly at higher doses (*p* < 0.05–0.0001), further supporting the antiproliferative potential of both preparations.

Many constituents identified in *P. scoparius*, particularly phenolic acids and essential oil components, may contribute to its potential antiproliferative activity. The cytotoxic effects observed in the present study may be associated with the high content of phenolic acids detected in the methanolic extract and the predominance of monoterpene hydrocarbons in the essential oil. These classes of compounds are widely reported to exhibit diverse biological activities, including antioxidant, anti-inflammatory, and antiproliferative effects. Among them, chlorogenic acid has been reported to exert significant preventive and therapeutic effects against several tumor types, including lung cancer [18], liver cancer [12], breast cancer [19], and colorectal cancer [20,21]. In addition, chlorogenic acid has been evaluated in Phase I clinical trials for the treatment of recurrent high-grade glioma, where it demonstrated favorable safety and tolerability [12]. Experimental studies have further shown that this compound can exert cytotoxic and antiproliferative effects, inhibit colony formation, invasion, and metastasis in HepG2 cells, and induce apoptosis in HT-29 cells through the activation of pro-apoptotic pathways and the suppression of anti-apoptotic protein expression.

Similarly, α-pinene has been reported to induce apoptosis in human gastric cancer cells by activating transcription factors involved in apoptotic signaling and modulating proteins associated with the MAPK (mitogen-activated protein kinase) pathway [22].

Taken together, the antiproliferative activity observed for *P. scoparius* may be associated with the contribution of major constituents such as chlorogenic acid and α-pinene, potentially enhanced by synergistic interactions among the different compounds present in the phytocomplex.

Cell viability data were analyzed using two-way ANOVA considering concentration and exposure time as independent variables, including the concentration × time interaction term. When significant interactions were detected, Tukey’s multiple comparison test was applied. Results are presented as mean ± SD and statistical significance is indicated in Figure 3.

### 2.3. Molecular Docking Insights into the Antiproliferative Mechanism of P. scoparius Phytoconstituents

**Figure 4:**
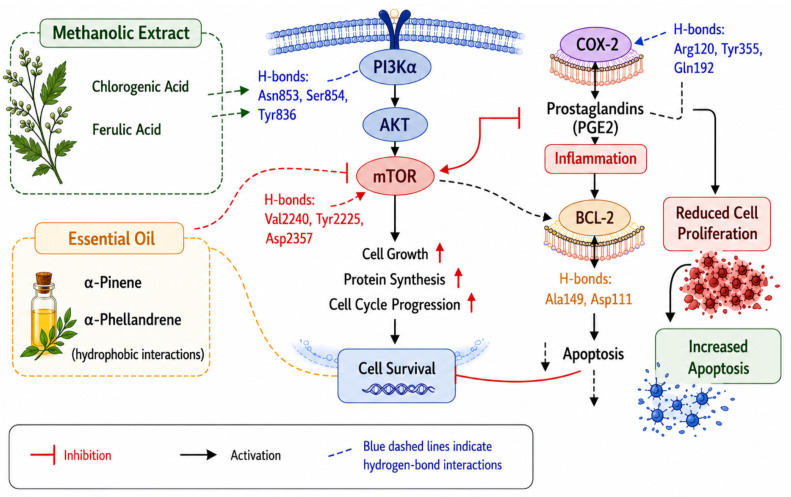
Proposed multitarget antiproliferative mechanism of *Pituranthos scoparius* extracts. The methanolic extract (chlorogenic acid and ferulic acid) and the essential oil (α-pinene and α-phellandrene) modulate key oncogenic pathways, including PI3Kα/AKT/mTOR, COX-2, and BCL-2. Molecular docking highlights hydrogen-bond interactions of phenolic compounds within catalytic or ATP-binding regions, contributing to inhibition of cell growth and survival signaling, reduced prostaglandin-mediated inflammation, and induction of apoptosis, ultimately resulting in decreased cancer cell proliferation. Inhibitory interactions are indicated in red, activation pathways in black, and blue dashed lines represent hydrogen-bond interactions between the identified phytochemicals and their molecular targets.

To mechanistically rationalize the antiproliferative effects observed in HepG2 and HT-29 cells, a structure-based molecular docking investigation was performed targeting key regulators of oncogenic growth, inflammatory signaling, and apoptosis. The selected targets were chosen based on their well-established involvement in hepatocellular carcinoma and colorectal cancer progression. PI3Kα and mTOR represent central components of the PI3Kα/AKT/mTOR signaling pathway, one of the most frequently dysregulated pathways in cancer, where it controls cell proliferation, metabolism, angiogenesis, and survival [23,24]. Cyclooxygenase-2 (COX-2) was selected because chronic inflammation is recognized as a major contributor to tumor development and progression, particularly in colorectal cancer [25]. B-cell lymphoma 2 (BCL-2) was included due to its pivotal role in regulating apoptosis and promoting resistance to programmed cell death in malignant cells [26]. Together, these targets provide a complementary representation of the major biological processes involved in cancer progression, namely proliferation, inflammation, and apoptosis [27]. The selected protein panel included PI3Kα (PDB ID: 7PG6) and mTOR (PDB ID: 4JSX), central nodes of the PI3Kα/AKT/mTOR proliferative axis; COX-2 (PDB ID: 5KIR), a major mediator of tumor-associated inflammation; and BCL-2 (PDB ID: 6GL8), an anti-apoptotic protein frequently overexpressed in solid tumors (Figure 4). Together, these targets provide a complementary representation of the major biological processes involved in cancer progression, namely proliferation, inflammation, and apoptosis, and were therefore considered suitable candidates for investigating the potential molecular basis of the antiproliferative effects observed for *P. scoparius*. This integrative panel reflects complementary pathways relevant to hepatocellular and colorectal carcinogenesis.

**Figure 5:**
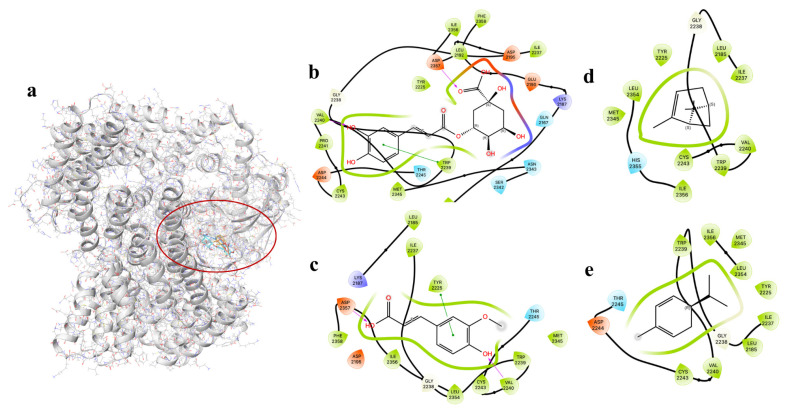
Structural representation and docking analysis of mTOR with selected ligands. (**a**) Overall structure of mTOR in complex with the four ligands, highlighting the binding pocket (red circle). Detailed 2D interaction diagrams of (**b**) chlorogenic acid, (**c**) ferulic acid, (**d**) α-pinene, and (**e**) α-phellandrene. In panel (**a**), the protein is shown as a gray cartoon, while the docked ligands are displayed as stick models. In the 2D interaction diagrams, residues are color-coded according to their physicochemical properties. Hydrogen bonds are indicated by magenta lines, π–π stacking interactions by green lines, and other non-covalent interactions are represented using the default Maestro color scheme.

**Figure 6:**
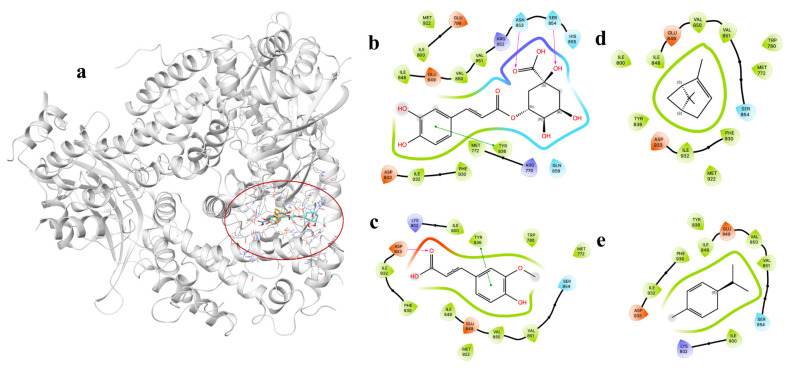
Structural representation and docking analysis of PI3Kα with selected ligands. (**a**) Overall structure of PI3Kα in complex with the four ligands, highlighting the binding pocket (red circle). Detailed 2D interaction diagrams of (**b**) chlorogenic acid, (**c**) ferulic acid, (**d**) α-pinene, and (**e**) α-phellandrene. In panel (**a**), the protein is shown as a gray cartoon, while the docked ligands are displayed as stick models. In the 2D interaction diagrams, residues are color-coded according to their physicochemical properties. Hydrogen bonds are indicated by magenta lines, π–π stacking interactions by green lines, and other non-covalent interactions are represented using the default Maestro color scheme.

Docking protocols were validated by redocking the co-crystallized inhibitors (Figures S1–S4), yielding RMSD values below 1.1 Å for all targets (PI3Kα: 0.58 Å; COX-2: 1.04 Å; mTOR: 0.91 Å; BCL-2: 0.67 Å), confirming the robustness and predictive reliability of the computational setup. Among all targets, mTOR showed the highest predicted affinity, particularly for chlorogenic acid (ChemPLP = 62.31, Table 5), which established seven hydrogen bonds (Figure 5b) involving Cys2243, Val2240, Tyr2225, Asp2357, and Asp2195 within or near the ATP-binding cleft. Ferulic acid also displayed relevant binding (ChemPLP = 57.46, Table 5), forming four hydrogen bonds in the catalytic pocket (Figure 5c). Given the pivotal role of mTOR in protein synthesis, cell-cycle progression, and metabolic regulation, its predicted inhibition is consistent with the pronounced reduction in cell viability observed in the MTT assay, especially at 48 h, suggesting time-dependent suppression of proliferative signaling. At the upstream level, both phenolic acids interacted favorably with PI3Kα (ChemPLP ≈ 55, Table 6), forming hydrogen bonds with hinge-region residues Asn853 and Ser854, as well as π-stacking interactions with Tyr836 (Figure 6b,c), indicative of ATP-competitive binding. Simultaneous targeting of PI3Kα and mTOR suggests potential dual blockade of the signaling cascade, thereby amplifying downstream inhibition of AKT activation and ultimately impairing tumor cell growth.

**Figure 7:**
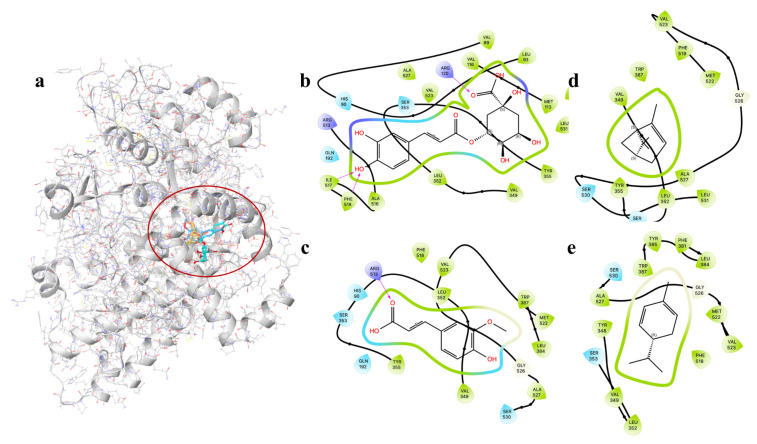
Structural representation and docking analysis of COX-2 with selected ligands. (**a**) Overall structure of COX-2 in complex with the four ligands, highlighting the binding pocket (red circle). Detailed 2D interaction diagrams of (**b**) chlorogenic acid, (**c**) ferulic acid, (**d**) α-pinene, and (**e**) α-phellandrene. In panel (**a**), the protein is shown as a gray cartoon, while the docked ligands are displayed as stick models. In the 2D interaction diagrams, residues are color-coded according to their physicochemical properties. Hydrogen bonds are indicated by magenta lines, π–π stacking interactions by green lines, and other non-covalent interactions are represented using the default Maestro color scheme.

**Figure 8:**
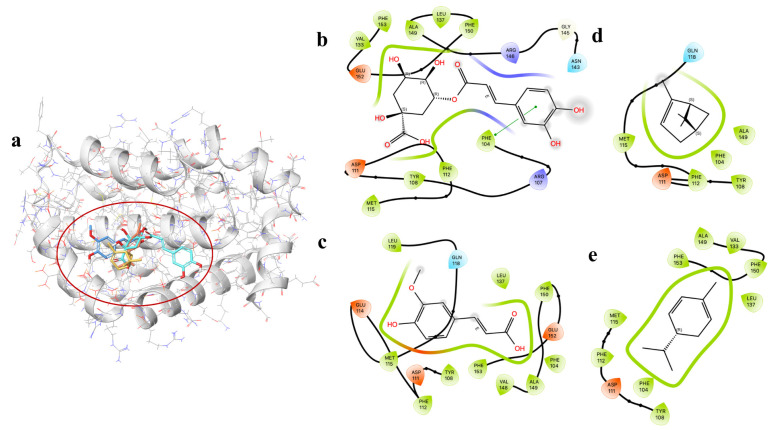
Structural representation and docking analysis of BCL-2 with selected ligands. (**a**) Overall structure of BCL-2 in complex with the four ligands, highlighting the binding pocket (red circle). Detailed 2D interaction diagrams of (**b**) chlorogenic acid, (**c**) ferulic acid, (**d**) α-pinene, and (**e**) α-phellandrene. In panel (**a**), the protein is shown as a gray cartoon, while the docked ligands are displayed as stick models. In the 2D interaction diagrams, amino acid residues are color-coded according to their physicochemical properties. Hydrogen bonds are indicated by magenta lines, while π–π stacking interactions are shown in green.

Chlorogenic acid also demonstrated moderate affinity toward COX-2 (ChemPLP = 50.86, Table 7), forming key anchoring hydrogen bonds with Arg120 and Tyr355 at the catalytic entrance (Figure 7b). These interactions may attenuate prostaglandin E2 synthesis, reducing inflammation-driven tumor progression. Additionally, interaction with BCL-2 (ChemPLP = 50.18, Table 8) through hydrogen bonding with Ala149 (Figure 8b) suggests partial interference with its anti-apoptotic function, potentially facilitating apoptosis induction.

Collectively, these data support a multitarget antiproliferative mechanism, where phenolic compounds may simultaneously modulate proliferative signaling, inflammation, and apoptosis pathways within a phytocomplex framework. The combined moderate-to-high affinity across several oncogenic nodes may contribute to a synergistic systems-level suppression rather than single-target inhibition, coherently explaining the progressive, concentration-dependent antiproliferative effect observed in HepG2 cells.

In contrast, α-pinene and α-phellandrene exhibited lower ChemPLP scores across all targets (36–47 range) and formed primarily hydrophobic interactions, without stable hydrogen bond networks within catalytic residues (Figure 5, Figure 6, Figure 7 and Figure 8). These findings suggest limited direct enzymatic inhibition of kinase or apoptotic proteins. However, α-phellandrene displayed slightly higher affinity toward COX-2 (ChemPLP = 45.63, Table 7) compared to α-pinene (39–40 range across targets), indicating possible weak modulation of inflammatory pathways. The antiproliferative effect of the essential oil observed in MTT assays is therefore unlikely to arise from strong ATP-competitive inhibition but may instead derive from alternative mechanisms typical of monoterpene-rich oils, including membrane perturbation, mitochondrial dysfunction, modulation of redox balance, and indirect apoptosis sensitization. Importantly, the GC–MS profile reveals that the essential oil is dominated by monoterpene hydrocarbons (α-pinene 38.90%, α-phellandrene 12.24%, sabinene 10.22%, *p*-cymene 8.80%), suggesting that the biological activity reflects a phytocomplex effect rather than a single compound action. Synergistic membrane destabilization and oxidative stress modulation may enhance intracellular susceptibility to growth suppression, explaining the strong antiproliferative response observed despite modest in silico binding affinities.

### 2.4. Machine Learning Integration

The machine learning–based target prediction analysis provided additional exploratory support for the multitarget mechanism suggested by the docking results (Table S2). Chlorogenic acid and *trans*-ferulic acid were predicted to interact with several protein classes involved in key cancer-related pathways, including kinases, oxidoreductases, and enzymes associated with inflammatory processes. Notably, predicted targets included proteins functionally related to the PI3Kα/AKT/mTOR signaling cascade and inflammatory mediators such as cyclooxygenases, in agreement with the docking results obtained for PI3Kα, mTOR, and COX-2. In contrast, α-pinene showed a lower number of predicted targets with lower probability scores, mainly associated with membrane-related proteins and enzymes involved in xenobiotic metabolism. This observation is consistent with its weaker binding affinity observed in docking simulations and supports the hypothesis that its biological activity may be mediated through indirect mechanisms, such as membrane perturbation or modulation of oxidative stress rather than specific enzyme inhibition. Overall, the machine learning–based target prediction analysis was used as a complementary exploratory tool to support the interpretation of the molecular docking results. The predicted targets should be considered hypothesis-generating and require further experimental validation. Nevertheless, the combined computational evidence suggests a potential multitarget pharmacological profile for *P. scoparius* extract, where phenolic compounds may contribute to the modulation of signaling pathways, while monoterpenes may exert complementary, non-specific effects.

## 3. Materials and Methods

### 3.1. Ethnopharmacological Background

*Pituranthos scoparius* is widely used in traditional Algerian medicine for the treatment of asthma, rheumatism, diabetes, and inflammatory conditions. These traditional applications suggest a potential pharmacological relevance related to oxidative stress and inflammation, which are closely associated with cancer development. Therefore, this study aims to provide a scientific validation of its traditional uses through an integrated phytochemical and biological investigation.

### 3.2. Plant Material Collection and Authentication

The aerial parts (stems and leaves) of *P. scoparius* were collected from Bouira (36° N, 4°54′7.2″ E), northern Algeria. The species naturally occurs in semi-arid Mediterranean ecosystems, typically characterized by steppe and rocky pasture vegetation, where it grows in sparse populations adapted to dry and nutrient-poor conditions. The soils of the region are generally calcareous to sandy–loamy in structure, with low organic matter content, which is consistent with the ecological preferences of *P. scoparius*. The plant was taxonomically authenticated by Pr. H. Hadj-Arab (Faculty of Biological Sciences, USTHB, Algeria). A voucher specimen (AB-71) was deposited in the herbarium of the Laboratory of Biology: Applications in Health and Environment, Université de M’sila, Algeria. The collected plant material was washed, air-dried at room temperature, ground into a fine powder, and stored until further use.

### 3.3. Preparation of Methanolic Extract

Fifty grams (50 g) of powdered aerial parts were extracted with 500 mL of 85% methanol/water (85/15) using a Soxhlet apparatus for 6 h. After extraction, the solution was filtered through Whatman No. 1 filter paper, and the filtrate was evaporated to dryness under reduced pressure using a rotary evaporator (Büchi 461, Büchi Labortechnik AG, Flawil, Switzerland) at 37 °C, yielding a dry methanolic extract as previously described [28]. The extract was stored at 4 °C until further analysis (yield: 3.18%).

### 3.4. Essential Oil Extraction

One hundred grams (100 g) of air-dried aerial parts were subjected to hydrodistillation using a Clevenger-type apparatus according to the European Pharmacopoeia procedure. The distillation was performed for 3 h and was stopped once no further significant increase in oil volume was observed [28]. The obtained essential oil was collected, dried over anhydrous sodium sulfate, and stored in amber vials at 4 °C until use (yield: 1.4%).

### 3.5. LC-MS/MS Phytochemical Analysis of P. scoparius Methanolic Extract

The methanolic extract of *P. scoparius* was analyzed to quantify bioactive compounds using an Agilent 6460 Triple Quadrupole LC/MS System (Agilent Technologies, Santa Clara, CA, USA). The LC system was equipped with a binary pump, an autosampler, and a thermostatted column compartment, coupled to a triple quadrupole mass spectrometer operating with an electrospray ionization (ESI) source. Briefly, 50 mg of methanolic extract was dissolved in 1 mL of an acetonitrile–methanol–water mixture (1:1:1, *v*/*v*/*v*) and vortexed until complete solubilization. Samples were further sonicated in an ultrasonic bath if necessary. Subsequently, 0.8 mL of hexane was added for defatting, followed by centrifugation at 7000 rpm for 5 min. The lower aqueous-methanolic phase was collected, diluted 1:4 (*v*/*v*), and filtered through a 0.25 μm membrane filter prior to analysis [29,30].

Mobile phases consisted of 0.1% formic acid and 5 mM ammonium formate in water (mobile phase A) and 0.1% formic acid in acetonitrile–methanol (mobile phase B). The elution gradient was as follows: 15% B (1–5 min), 25% B (6–15 min), 75% B (16–25 min), followed by re-equilibration at 15% B (26–40 min). The injection volume was 4.0 μL, and the flow rate was 0.4 mL/min. Chromatographic separation was achieved on a Poroshell 120 EC-C18 column (50 mm × 4.6 mm i.d., 2.7 μm particle size) maintained at 30 °C. Mass spectrometric analysis of 33 phenolic and flavonoid compounds was performed in multiple reaction monitoring (MRM) mode. Optimized MRM transitions, dwell times, fragmentor voltages, and collision energies for each analyte are summarized in Table 2. Electrospray ionization was applied in both positive and negative modes depending on compound ionization characteristics. Quantification was achieved using external calibration curves prepared from commercially available reference standards (≥95% purity; suppliers listed in Supplementary Information). Regression equations and correlation coefficients (R^2^) for each analyte are provided in Table 2. Limits of detection (LOD) and quantification (LOQ) were calculated according to the equations LOD = 3.3 × (σ/S) and LOQ = 10 × (σ/S), where S represents the slope of the calibration curve and σ is the standard deviation estimated from regression data; the complete set of values is reported in Table S1 (Supporting Information).

### 3.6. GC–MS/MS Analysis of Phytochemical Constituents of P. scoparius Essential Oil

GC–MS/MS analysis was performed using an Agilent 7890 gas chromatograph coupled to an Agilent 7000 B Triple Quadrupole mass spectrometer (Agilent Technologies, Santa Clara, CA, USA). The instrument was equipped with an automated liquid/headspace sampler and fitted with an HP-5ms capillary column (30 m × 0.25 mm × 0.25 μm film thickness), specifically an HP-5ms Ultra Inert capillary column. Electron ionization (EI) at 70 eV was employed, and detection was performed in full-scan mode over a mass range of *m*/*z* 35–1000, alternatively reported as *m*/*z* 50–550 with a solvent delay of 3.0 min [31]. Additional details regarding fatty acid analysis have been previously described [32]. Helium was used as the carrier gas at a flow rate of 1.0 mL/min, and the injector temperature was maintained at 250 °C with injections performed in split mode at a split ratio of 20:1. The oven temperature program was as follows: initial temperature of 50 °C held for 2 min, increased to 140 °C at 3 °C/min, then to 220 °C at 4 °C/min, and finally to 270 °C at 5 °C/min. Headspace sampling conditions were set at 130 °C for the oven, 140 °C for the loop, and 145 °C for the transfer line, with a vial equilibration time of 30 min, while the transfer line temperature has also been reported as maintained at 250 °C. For each run, 0.1 g of sample was introduced into a sealed headspace vial, equilibrated under the specified conditions, and automatically injected into the GC–MS/MS system for analysis. Compound identification was performed by comparing the obtained mass spectra with those available in the NIST spectral library and by calculating retention indices relative to a series of n-alkane standards (C7–C30) analyzed under the same chromatographic conditions [29].

### 3.7. Cell Culture and In Vitro Cytotoxicity Assay

Human colorectal carcinoma (HT-29) and hepatocellular carcinoma (HepG2) cell lines were obtained from the Cell Culture Laboratory, Research and Application Laboratory, Iğdır University. Cells were maintained in RPMI-1640 medium supplemented with 10% (*v*/*v*) heat-inactivated fetal bovine serum (FBS) and 1% (*v*/*v*) L-glutamine at 37 °C in a humidified atmosphere containing 5% CO_2_.

Cells were seeded into 96-well plates at a density of 5 × 10^4^ cells/mL and allowed to adhere for 24 h to reach the logarithmic growth phase. The methanolic extract and essential oil of *P. scoparius* were dissolved in DMSO to prepare 100 mg/mL stock solutions. Working concentrations ranging from 100 to 1.56 μg/mL were obtained by serial dilution in culture medium, ensuring that the final DMSO concentration did not exceed 0.1% to avoid solvent-related cytotoxicity. Cells were treated with these concentrations for 24 and 48 h under standard culture conditions.

Cell viability was assessed using the MTT assay. After treatment, the medium was replaced with fresh medium containing 10% MTT solution (5 mg/mL in PBS) and incubated for 3 h at 37 °C in the dark. Formazan crystals were dissolved in DMSO, and absorbance was measured at 570 nm using a microplate reader [33]. Each treatment was performed in triplicate, and experiments were repeated in three independent runs. Cell viability was calculated as a percentage of untreated controls using the formula:

Cell viability% = (mean absorbance of treated cells/mean absorbance of control cells) × 100%

Dose–response curves were generated from these values to determine the IC_50_ for each extract [34].

### 3.8. Molecular Docking Study of Selected Metabolites from P. scoparius Methanolic Extract and Essential Oil

The three-dimensional structures of the selected protein targets were retrieved from the Protein Data Bank (PDB): PI3Kα (PDB ID: 7PG6), mTOR kinase domain (PDB ID: 4JSX), COX-2 (PDB ID: 5KIR), and BCL-2 (PDB ID: 6GL8). Proteins were selected based on their high-resolution structures and the presence of a co-crystallized ligand within the catalytic site.

Prior to docking, protein structures were prepared by removing unnecessary chains (e.g., Chain B) using Molecular Explorer, while retaining water molecules directly involved in ligand interactions. Hydrogen atoms were added, and proper protonation states were assigned using the Docking Wizard panel. The binding site was defined based on the position of the co-crystallized ligand, with a cavity radius of 6 Å. The native ligand was extracted and redocked to validate the docking protocol, yielding root mean square deviation (RMSD) values of 1.04 Å (PI3Kα and COX-2), 0.91 Å (mTOR), and 0.67 Å (BCL-2), confirming protocol reliability (RMSD < 2.0 Å). The co-crystallized ligand was retained as a reference for subsequent docking comparisons. Ligand structures, including chlorogenic acid, ferulic acid, α-pinene, α-phellandrene, and other selected essential oil constituents, were downloaded in .sdf format from the PubChem database. Ligand preprocessing in GOLD software (Cambridge Crystallographic Data Centre, CCDC, version 2024.2.0, Cambridge, UK) included atom typing, hydrogen addition, lone pair generation, identification of hydrogen bond donors and acceptors, and aromatic fragment recognition. Docking simulations were performed using the ChemPLP scoring function to rank ligand poses. Predicted binding affinities were expressed as PLP.fitness scores, reflecting hydrogen bonding, metal interactions, and steric complementarity rather than absolute thermodynamic ΔG values. Pose accuracy was validated through redocking of co-crystallized ligands, with RMSD values consistently below 1.1 Å. For each ligand, the top three poses were retained, applying a clustering RMSD tolerance of 1.5 Å. Protein–ligand interactions, including hydrogen bonds and hydrophobic contacts, were analyzed using Hermes (version 2024.2.0). All calculations were performed on a macOS system using an Apple MacBook Pro with an M2 Pro processor running macOS Ventura 13.2 (Apple Inc., Cupertino, CA, USA).

### 3.9. Machine Learning–Based Target Prediction

To complement the experimental and docking results and to strengthen the identification of potential antiproliferative targets, a machine learning–based target prediction analysis was performed to identify potential biological targets of the major compounds detected in *P. scoparius*. The chemical structures of selected metabolites, including chlorogenic acid, *trans*-ferulic acid, and α-pinene, were retrieved from the PubChem database in SMILES format. Target prediction was carried out using the SwissTargetPrediction platform (Swiss Institute of Bioinformatics, Lausanne, Switzerland), which applies a combination of 2D and 3D similarity-based machine learning algorithms trained on a large dataset of bioactive molecules to estimate the probability of interaction with human protein targets (Table S2). Predictions were restricted to Homo sapiens, and only targets with a probability score ≥0.1 were considered for further analysis. The predicted targets were then compared with those selected for molecular docking (PI3Kα, mTOR, COX-2, and BCL-2) to evaluate the consistency between machine learning predictions and structure-based docking results.

### 3.10. Statistical Analysis

Data are presented as mean ± standard deviation (SD). Cell viability following exposure to different extract concentrations for 24 and 48 h was analyzed using two-way analysis of variance (ANOVA), with concentration and time as independent factors. Tukey’s post hoc test was applied for multiple comparisons between groups. Statistical analyses were performed using GraphPad Prism^®^ version 9.0.0 (GraphPad Software, Boston, MA, USA), and a *p*-value < 0.05 was considered statistically significant.

## 4. Conclusions

In this study, the chemical composition of *Pituranthos scoparius* extract and essential oil was comprehensively analyzed, revealing a diverse range of bioactive compounds, including phenolic acids, flavonoids, and monoterpenes such as α-pinene and sabinene. LC–MS/MS analysis identified chlorogenic acid (191.4 µg/g) and *trans*-ferulic acid (96.2 µg/g) as the predominant constituents of the methanolic extract, while GC–MS/MS analysis revealed an α-pinene–rich essential oil (38.90%) followed by α-phellandrene (12.24%) and sabinene (10.22%). The evaluation of cytotoxic activity demonstrated that *P. scoparius* exhibits moderate but significant antiproliferative activity, consistent with the multitarget nature of phytocomplexes against HT-29 (colorectal cancer) and HepG2 (liver cancer) cell lines, with a time-dependent enhancement of activity at 48 h. The essential oil showed marked cytotoxicity toward HT-29 cells (IC_50_ = 35.4 ± 4.57 µg/mL), whereas the methanolic extract displayed selective activity against HepG2 cells (IC_50_ = 95.16 ± 10.86 µg/mL), indicating a differential, cell-specific response. Molecular docking studies provided complementary insights, highlighting favorable binding interactions of chlorogenic acid and *trans*-ferulic acid with key oncogenic targets, particularly mTOR (ChemPLP score up to 69.31) and PI3Kα (ChemPLP score up to 55.28), as well as relevant interactions with COX-2 and BCL-2. These in silico results, together with machine learning–based target prediction analysis, consistently indicate a multitarget modulation of proliferative, inflammatory, and anti-apoptotic signaling pathways, strengthening the mechanistic interpretation of the observed antiproliferative effects. Overall, this study provides an integrated approach combining phytochemical profiling, in vitro cytotoxicity, molecular docking, and machine learning–based target prediction to describe a phytocomplex-driven multitarget antiproliferative potential of *P. scoparius*. Further investigations in advanced biological models would provide additional insight into the molecular mechanisms and help translate these findings into potential therapeutic applications.

Nevertheless, several limitations of this study should be acknowledged. First, no normal (non-tumorigenic) cell lines were included to evaluate the selectivity and safety profile of the extracts. Second, the proposed molecular mechanisms derived from docking and in silico predictions were not validated through biochemical or molecular biology experiments. Third, the crude extract and essential oil were not subjected to bio-guided fractionation; therefore, the specific active fractions or compounds responsible for the observed effects remain unidentified. Finally, the potential synergistic or individual effects of isolated phytochemicals were not experimentally investigated. Further investigations in advanced biological models would provide additional insight into the molecular mechanisms and help translate these findings into potential therapeutic applications.

## Figures and Tables

**Table 1:** Identification and quantification of specialized metabolites in the methanolic extract of *P. scoparius* by LC–MS/MS analysis using both negative and positive electrospray ionization modes (ESI^−^ and ESI^+^). The content of each metabolite is expressed as µg/g of extract.

Peak ^a^	Tentative Identification ^b^	R.t. (min)	Biochemical Class	Mw (g/mol)	Final Concentration (µg/g Extract)
1	Gallic acid	1.94	Phenolic acid	170.12	ND
2	Epigallocatechin	1.98	Flavanol	306.27	2.0 ± 0.1
3	Gentisic acid	2.44	Phenolic acid	154.12	18.9 ± 0.8
4	Chlorogenic acid	2.57	Phenolic acid	354.31	191.4 ± 4.8
5	Catechin	2.58	Flavanol	290.27	ND
6	Caffeic Acid	2.80	Phenolic acid	180.16	2.6 ± 0.1
7	Vanillic acid	2.98	Phenolic acid	168.15	39.3 ± 1.2
8	Syringic acid	3.40	Phenolic acid	198.17	14.8 ± 0.5
9	Rutin	3.69	Flavonol glycoside	610.52	7.7 ± 0.3
10	Isoquercitrin	4.35	Flavonol glycoside	464.38	ND
11	*p*-Coumaric acid	4.50	Phenolic acid	164.16	9.1 ± 0.4
12	Polydatin	4.53	Stilbene glycoside	390.38	ND
13	Hydroxybenzaldehyde	4.61	Phenolic aldehyde	122.12	1.0 ± 0.1
14	Sinapic acid	4.75	Phenolic acid	224.21	16.7 ± 0.6
15	*trans*-Ferulic acid	4.98	Phenolic acid	194.18	96.2 ± 2.5
16	Vanillin	5.83	Phenolic aldehyde	152.15	61.8 ± 1.9
17	Taxifolin	5.90	Flavanonol	304.25	ND
18	Salicylic Acid	7.29	Phenolic acid	138.12	12.1 ± 0.4
19	*o*-coumaric acid	7.84	Phenolic acid	164.16	ND
20	Baicalin	7.97	Flavone glycoside	446.36	ND
21	Protocatehuic ethyl ester	8.36	Phenolic acid	182.17	ND
22	Protocatechuic acid	8.52	Phenolic acid	154.12	ND
23	Quercetin	10.02	Flavonol	302.24	ND
24	*trans*-Cinnamic acid	10.87	Phenylpropanoid	148.16	5.8 ± 0.2
25	Naringenin	11.54	Flavanone	272.25	0.5 ± 0.1
26	Kaempferol	12.26	Flavonol	286.24	8.8 ± 0.3
27	Morin	11.93	Flavonol	302.24	1.9 ± 0.1
28	7-Hydroxyflavone	12.13	Flavone	238.24	ND
29	Chrysin	13.67	Flavone	254.24	ND
30	Luteolin	13.84	Flavone	286.24	ND
31	Biochanin A	13.82	Isoflavone	284.26	0.7 ± 0.1
32	5-Hydroxyflavone	15.73	Flavone	238.24	ND
33	Diosgenin	20.30	Steroidal saponin	414.63	2.7 ± 0.1
Total phenolic acids					401.0 ± 10.8
Total hydroxycinnamic acids					321.7 ± 8.8
Total flavonoids					21.6 ± 0.8
Total polyphenols					422.6 ± 8.9

^a^ See Figure 1 for peak assignment, ^b^ Retention time (r.t.) obtained using a Poroshell 120 EC-C18 column (50 mm × 4.6 mm i.d., 2.7 µm) with 2.5% formic acid; mean of three replicates, c Identification carried out by mass spectrometric data (MS/MS), Values are expressed as mean ± SD (*n* = 3). ND = not detected.

**Table 2:** MRM parameters and calibration data for the quantification of 33 phenolic and flavonoid compounds by LC–MS/MS.

Compounds	Precursor Ion (*m*/*z*)	Product Ion (s) (*m*/*z*)	Dwell (ms)	Fragmentor (V)	CE (V)	P/N	R^2^	Calibration Curves
Gallic acid	168.9	124.9	9	100	12	N	0.9936	y = 103.892941x − 732.464249
Epigallocatechin	307	139	9	99	8	P	0.9989	y = 30.570814x − 523.264315
Gentisic acid	152.8	108.9, 108	9	100	13	N	0.9979	y = 23.823434x − 2195.988700
Chlorogenic acid	353	191	9	110	12	N	0.9986	y = 79.725061x − 112.913598
Catechin	291	165, 139, 123	9	80	12	P	0.9946	y = 66.879735x + 705.284086
Caffeic acid	178.9	135	9	80	16	N	0.9988	y = 244.717806x + 763.242916
Vanillic acid	167.1	152, 123.1, 107.7	9	96	12	N	0.9986	y = 3.094039x − 195.176594
Syringic acid	199	154.9, 140.2	9	85	6	P	0.9991	y = 57.460290x − 353.313587
Rutin	609.1	300.2	9	185	40	N	0.9977	y = 49.187937x + 576.491215
Isoquercitrin	464.9	302.8	9	95	8	P	0.9988	y = 74.819750x + 3466.943231
*p*-Coumaric acid	163.1	119.2	9	85	12	N	0.9959	y = 88.298152x
Polydatin	391	229	9	80	8	P	0.9971	y = 90.617974x
Hydroxybenzaldehyde	121	92	9	110	24	N	0.9972	y = 285.932691x − 864.464641
Sinapic acid	224.9	206.9, 174.8	9	90	3	P	0.9997	y = 364.976677x + 2936.434090
*trans*-Ferulic acid	195.1	176.9, 144.7	9	75	10	P	0.9995	y = 137.603071x − 1364.653093
Vanillin	152.9	124.9, 93.1, 65.2	9	65	13	P	0.9988	y = 150.584236x + 481.186284
Taxifolin	303	284.8, 124.7	9	144	6	N	0.9945	y = 68.774190x + 523.230880
Salicylic acid	137	93.1	38	85	18	N	0.9982	y = 78.344927x − 1702.143390
o-Coumaric acid	162.8	119	38	66	13	N	0.9999	y = 33.637841x − 192.193627
Baicalin	447	270.9, 112.5	38	130	16	P	0.9991	y = 43.366428x − 142.647220
Protocatechuic ethyl ester	181	152.9, 107.9	38	110	20	N	0.9971	y = 315.693022x + 4389.808066
Protocatechuic acid	154.9	136.4, 111, 93.1	38	118	11	P	0.9978	y = 278.319907x − 4951.703456
Quercetin	302.8	152.9, 136.9	18	125	34	P	0.9931	y = 12.062621x + 149.979848
*trans*-Cinnamic acid	149	131.1, 103	18	75	6/10	P	0.9984	y = 24.887968x − 250.053648
Naringenin	270.9	150.8, 119.1	18	130	26/24	N	0.9995	y = 74.163906x − 339.741696
Kaempferol	287	212.9, 164.7, 152.7, 120.9	18	120	34	P	0.9969	y = 26.752168x + 474.681696
Morin	302.9	136.8, 153	18	145	32	P	0.9965	y = 335.860146x − 6474.081178
7-Hydroxyflavone	238.9	137.1	18	170	30	P	0.9971	y = 1084.261320x + 8992.232794
Chrysin	255	102.9, 153	48	155	30	P	0.9984	y = 268.266601x + 4129.475946
Luteolin	285	133.1	48	132	36	P	0.9948	y = 37.598664x + 749.712439
Biochanin A	284.9	151.9	48	170	28	P	0.9998	y = 687.526959x − 4833.287362
5-Hydroxyflavone	238.9	165	48	170	36	P	0.9983	y = 336.096989x − 2537.475857
Diosgenin	415.3	271.1, 253	48	110	16	P	0.9982	y = 109.547642x − 2991.919601

**Table 3:** Chemical composition of the essential oil from the aerial parts of *P. scoparius* identified by GC–MS/MS.

Peak	Compound	RT (min)	RI (NIST List.)	RI (Calc.)	%
1	α-Thujene	10.15	929	921	3.48
2	α-Pinene	10.45	937	929	38.90
3	Camphene	11.03	952	945	0.13
4	Sabinene	12.08	974	969	10.22
5	β-Pinene	12.21	979	972	7.20
6	β-Myrcene	12.83	991	985	1.66
7	α-Phellandrene	13.41	1005	996	12.24
8	3-Carene	13.67	1011	1001	1.03
9	α-Terpinene	13.96	1017	1009	0.37
10	*p*-Cymene	14.32	1022	1018	8.80
11	β-Terpinene	14.52	1028	1023	3.89
12	β-Ocimene	14.93	1037	1033	5.08
13	γ-Terpinene	15.88	1060	1054	1.07
14	Isoterpinolene	17.23	1086	1082	0.25
15	Neo-allo-ocimene	19.10	1131	1122	0.34
16	4-terpineol	21.35	1182	1172	2.14
17	α-Terpineol	21.96	1190	1184	0.15
18	Carveol	22.47	1219	1194	0.29
19	Germacrene D	34.35	1481	1476	0.56
20	tau-Muurolol	39.77	1642	1637	0.38
21	*cis*-Butylidenephthalide	40.69	1678	1667	0.56
22	*trans*-Ligustilide	42.52	1810	1731	1.27

**Table 4:** IC_50_ values of the methanolic extract and essential oil of *P. scoparius* against HT-29 and HepG2 cancer cell lines after 24 and 48 h of treatment.

	HepG2		HT-29	
	24 h	48 h	24 h	48 h
ME	*	95.16 ± 10.86	*	*
EO	110.70 ± 9.09	54.89 ± 11.24	80.94 ± 10.55	35.4 ± 4.57
5-Fluorouracil (5-FU, positive control)	62.77 ± 6.80	9.89 ± 2.48	55.65 ± 3.19	17.94 ± 2.33

(Values are expressed as mean ± standard deviation. IC_50_ values indicate the concentration at which 50% of cell viability is inhibited). IC_50_ values were calculated by nonlinear regression. The symbol (*) now indicates that IC_50_ values could not be determined within the tested concentration range.

**Table 5:** Molecular docking results of the main metabolites identified in the methanolic extract and essential oil of *P. scoparius* toward mTOR kinase.

Ligand	ChemPLP Score	No. of H-Bonds	Key Residues	Other Interactions
Chlorogenic acid	62.31	6	Cys2243, Val2240, Tyr2225, Asp2357, Asp2195	Hydrophobic
Ferulic acid	57.46	4	Val2240, Tyr2225, Asp2357, Asp2195	Hydrophobic
α-pinene	38.08	-	-	Hydrophobic
α-phellandrene	47.3	-	-	Hydrophobic

Docking simulations were performed using GOLD software 2024.2.0 (build 415170) (CCDC). The binding site was defined based on the co-crystallized ligand in mTOR (PDB ID: 4JSX). ChemPLP was used as a scoring function. The docking protocol was validated by redocking the co-crystallized inhibitor Torin-2 into the mTOR kinase binding site (RMSD of 0.91 Å).

**Table 6:** Molecular docking results of the main metabolites identified in the methanolic extract and essential oil of *P. scoparius* toward PI3Kα.

Ligand	ChemPLP Score	No. of H-Bonds	Key Residues	Other Interactions
Chlorogenic acid	55.28	6	Asn853, Ser854, His855	Pi-stacking Tyr836
Ferulic acid	55.16	1	Asp933, Val851	Pi-stacking Tyr836
α-pinene	42.72	-	-	Hydrophobic
α-phellandrene	40.03	-	-	Hydrophobic

Docking simulations were performed using GOLD software (CCDC). The binding site was defined based on the co-crystallized ligand in PI3Kα (PDB ID: 7PG6). ChemPLP was used as a scoring function. The docking protocol was validated by redocking the co-crystallized inhibitor alpelisib into the PI3Kα binding site (RMSD of 0.58 Å).

**Table 7:** Molecular docking results of the main metabolites identified in the methanolic extract and essential oil of *P. scoparius* toward COX2.

Ligand	ChemPLP Score	No. of H-Bond	Key Residues	Other Interactions
Chlorogenic acid	50.86	5	Arg120, Tyr355, Gln192, Ile517, Phe518	Hydrophobic
Ferulic acid	51.95	3	Arg513, Gln192, Ser530	Hydrophobic
α-pinene	39.11	-	-	Hydrophobic
α-phellandrene	45.63	-	-	Hydrophobic

Docking simulations were performed using GOLD software (CCDC). The binding site was defined based on the co-crystallized ligand in COX2 (PDB ID: 5KIR). ChemPLP was used as a scoring function. The docking protocol was validated by redocking the co-crystallized inhibitor rofecoxib (Vioxx) into the COX-2 binding site (RMSD of 1.04 Å).

**Table 8:** Molecular docking results of the main metabolites identified in the methanolic extract and essential oil of *P. scoparius* toward BCL-2.

Ligand	ChemPLP Score	No. of H-Bonds	Key Residues	Other Interactions
Chlorogenic acid	50.18	2	Ala149, Phe104	Hydrophobic
Ferulic acid	43.42	3	Ala149, Gln118, Asp111	Hydrophobic
α-pinene	36.45	-	-	Hydrophobic
α-phellandrene	38.88	-	-	Hydrophobic

Docking simulations were performed using GOLD software (CCDC). The binding site was defined based on the co-crystallized ligand in BCL-2 (PDB ID: 6GL8). ChemPLP was used as a scoring function. The docking protocol was validated by redocking the co-crystallized inhibitor BCL-201into the BCL-2 kinase binding site (RMSD of 0.67 Å).

## Data Availability

The data presented in this study are available within the article and Supplementary Materials. Additional data are available from the corresponding author upon reasonable request.

## Supplementary Materials

The following supporting information can be downloaded at: https://www.mdpi.com/article/10.3390/molecules31152561/s1, Table S1: Limits of detection (LOD) and quantification (LOQ) for phenolic and flavonoid compounds determined by LC–MS/MS; Table S2: Predicted molecular targets of selected phytoconstituents of *Pituranthos scoparius* obtained by SwissTargetPrediction; Figure S1: Validation by re-docking between alpelisib and PI3Kα; Figure S2: Validation by re-docking between Torin-2 and mTOR; Figure S3: Validation by re-docking between Vioxx and COX-2; Figure S4: Validation by re-docking between BCL201 and BCL-2.

## Abbreviations

The following abbreviations are used in this manuscript:

ANOVA	Analysis of variance
BCL-2	B-cell lymphoma 2 protein
COX-2	Cyclooxygenase-2 (prostaglandin-endoperoxide synthase 2)
DMSO	Dimethyl sulfoxide
EO	Essential oil
ESI	Electrospray ionization
FBS	Fetal bovine serum
GC–MS/MS	Gas chromatography–tandem mass spectrometry
IC_50_	Half-maximal inhibitory concentration
LC–MS/MS	Liquid chromatography–tandem mass spectrometry
LOD	Limit of detection
LOQ	Limit of quantification
ME	Methanolic extract
ML	Machine learning
MRM	Multiple reaction monitoring
mTOR	Mechanistic target of rapamycin
MTT	3-(4,5-dimethylthiazol-2-yl)-2,5-diphenyltetrazolium bromide assay
PDB	Protein Data Bank
PI3Kα	Phosphoinositide 3-kinase alpha isoform
RMSD	Root mean square deviation.
