# Supplementary material for: Multitarget Antiproliferative Activity of Pituranthos scoparius: An Integrated Phytochemical, Biological and Computational Insights into Key Oncogenic Pathways
Source: Molecules. 2026 Jul 23;31(15):2561. doi: 10.3390/molecules31152561 (PMC13468436; doi:10.3390/molecules31152561)
Supplement: Supplementary file 1 [file molecules-31-02561-s001.zip › molecules-4363779-supplementary.pdf]

Supporting information to the manuscript titled:

Multitarget Antiproliferative Activity of *Pituranthos scoparius*: An Integrated Phytochemical, Biological and Computational Insights into Key Oncogenic Pathways

Sarra Chabane [1,2], Amel Boudjelal [2,3,*], Luana Pulvirenti [4,*], Aslı Yıldırım Kocaman [5], Ibrahim Demirtas [6], İlyas Yıldız [7], Fatih Gül [8], Süleyman Muhammed Çelik [9] and Amrane Abdeltif [10]

[1]*Department of Veterinary, Faculty of Sciences, University of M'sila, M'sila 28000, Algeria*
[2] *Laboratory of Biology: Applications in Health and Environment, University of M'sila, M'sila 28000, Algeria*
[3] *Department of Microbiology and Biochemistry, Faculty of Sciences, University of M'sila, M'sila 28000, Algeria*
[4] *Institute of Biomolecular Chemistry (ICB), National Research Council of Italy, Via Paolo Gaifami 18, 95126 Catania, Italy*
[5] *Department of Health Services, Vocational School of Higher Education for Healthcare Services, Igdir University, 76000 Igdir, Türkiye*
[6] *Department of Pharmaceutical Chemistry, Faculty of Pharmacy, Ondokuz Mayıs University, 55270 Samsun, Türkiye*
7 Ecole Nationale Supérieure de Chimie de Rennes, CNRS, L'Institut des Sciences Chimiques de Rennes (ISCR–UMR 6226), University of Rennes, 35000 Rennes, France
[8] *Department of Molecular Biology and Genetics, Tokat Gaziosmanpasa University, 60000 Tokat, Türkiye*
[9] *Research Laboratories and Research Application Center (ALUM), Igdir University, 76000 Igdir, Türkiye*
[10] *Department of Medical Services and Techniques, School of Health Services, Igdir University, 76000 Igdir, Türkiye*

* Correspondence: amel.boudjelal@univ-msila.dz (A.B.); luana.pulvirenti@cnr.it (L.P.);
Tel.: +213-540186575 (A.B.); +39-0957338111 (L.P.); Fax: +213-35-13-38-59 (A.B.); +39-0957338206 (L.P.)

Contents of Supporting Information

- **Table S1**. Limits of detection (LOD) and quantification (LOQ)
- **Table S2.** Target prediction
- **Figure S1**. Docking Validation method of PI3Kα
- **Figure S2.** Docking Validation method of mTOR
- **Figure S3.** Validation method of COX-2
- **Figure S4**. Validation method of BCL-2

Table S1. Limits of detection (LOD) and quantification (LOQ) for phenolic and flavonoid compounds determined by LC–MS/MS

**Compound**	**Slope (S)**	**R^2**	**LOD (µg/mL)**	**LOQ (µg/mL)**
Gallic acid	103.893	0.99357	0.0212	0.0643
Epigallocatechin	30.571	0.99891	0.0036	0.0109
Chlorogenic acid	79.725	0.99863	0.0045	0.0137
Catechin	66.880	0.99462	0.0178	0.0538
Gentisic acid	23.823	0.99791	0.0069	0.0209
Caffeic acid	244.718	0.99884	0.0038	0.0116
Syringic acid	57.460	0.99907	0.0031	0.0093
Vanillic acid	3.094	0.99857	0.0047	0.0143
Rutin	49.188	0.99772	0.0075	0.0228
Isoquercitrin	74.820	0.99876	0.0041	0.0124
p-Coumaric acid	88.298	0.99592	0.0135	0.0408
Sinapic acid	364.977	0.99969	0.0010	0.0031
Vanillin	150.584	0.99880	0.0040	0.0120
trans-Ferulic acid	137.603	0.99951	0.0016	0.0050
Taxifolin	68.774	0.99448	0.0182	0.0552
Salicylic acid	78.345	0.99820	0.0059	0.0180
o-Coumaric acid	33.638	0.99990	0.0003	0.0010
Baicalin	43.366	0.99914	0.0028	0.0086
Protocatechuic acid	278.320	0.99777	0.0074	0.0223
Quercetin	12.063	0.99309	0.0227	0.0691
Trans-cinnamic acid	24.888	0.99844	0.0052	0.0156
Naringenin	74.164	0.99948	0.0017	0.0052
Kaempferol	26.752	0.99695	0.0101	0.0305
Morin	335.860	0.99650	0.0116	0.0350
7-Hydroxyflavone	1084.261	0.99712	0.0095	0.0288
Chrysin	268.267	0.99843	0.0052	0.0157
Luteolin	37.599	0.99480	0.0172	0.0520
Biochanin A	687.527	0.99980	0.0007	0.0020
5-Hydroxyflavone	336.097	0.99834	0.0055	0.0166
Diosgenin	109.548	0.99821	0.0059	0.0179

LOD and LOQ values were calculated according to the equations: $LOD = 3.3 \times (\sigma/S)$ and $LOQ = 10 \times (\sigma/S)$, where S is the slope of the calibration curve. The standard deviation (σ) was estimated from the linear regression as $\sigma \approx (1 - R^2) \times S$, based on the correlation coefficient (R^2) of each calibration curve. All values are expressed in µg/mL.

Table S2. Predicted molecular targets of selected phytoconstituents of *Pituranthos scoparius* obtained by SwissTargetPrediction

Compound	Predicted target*	Target class	Probability	Biological relevance
Chlorogenic acid	PI3K family	Kinase	High	Cell proliferation / survival
Chlorogenic acid	mTOR pathway-related proteins	Kinase	Moderate–High	Protein synthesis / growth
Chlorogenic acid	Cyclooxygenases (COX-1/COX-2)	Oxidoreductase	Moderate	Inflammation
Chlorogenic acid	MAP kinases	Kinase	Moderate	Signal transduction
Ferulic acid	PI3K-related kinases	Kinase	Moderate	Cell signaling
Ferulic acid	COX enzymes	Oxidoreductase	Moderate	Inflammatory response
Ferulic acid	Tyrosine kinases	Kinase	Low–Moderate	Proliferation pathways
α-Pinene	Cytochrome P450 enzymes	Enzyme	Low	Xenobiotic metabolism
α-Pinene	Membrane-associated proteins	Various	Low	Membrane interaction
α-Pinene	Oxidoreductases	Enzyme	Low	Redox balance

*Predicted molecular targets of selected phytoconstituents identified in Pituranthos scoparius using SwissTargetPrediction. Target prediction was performed based on 2D and 3D similarity-based machine learning algorithms. Only targets with significant probability scores were considered. Target classes and biological relevance were assigned based on UniProt and literature data.

Figure S1. Validation by re-docking between alpelisib and PI3Kα

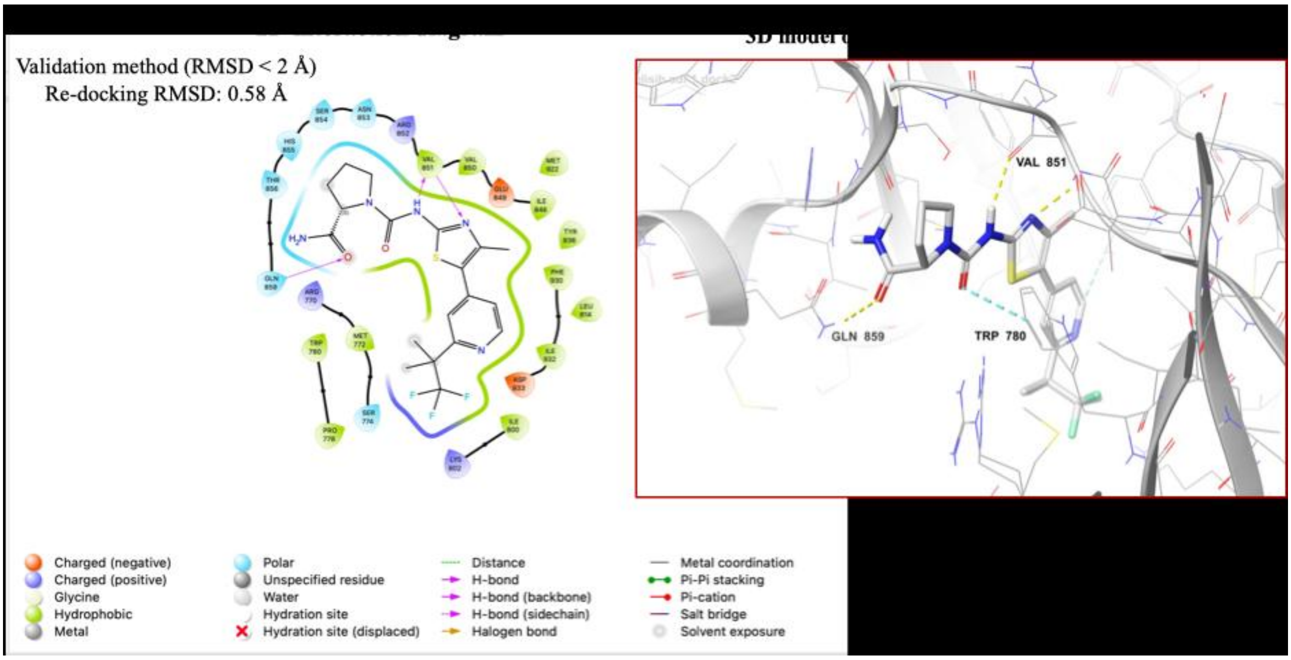

Figure S2. Validation by re-docking between Torin-2 and mTOR

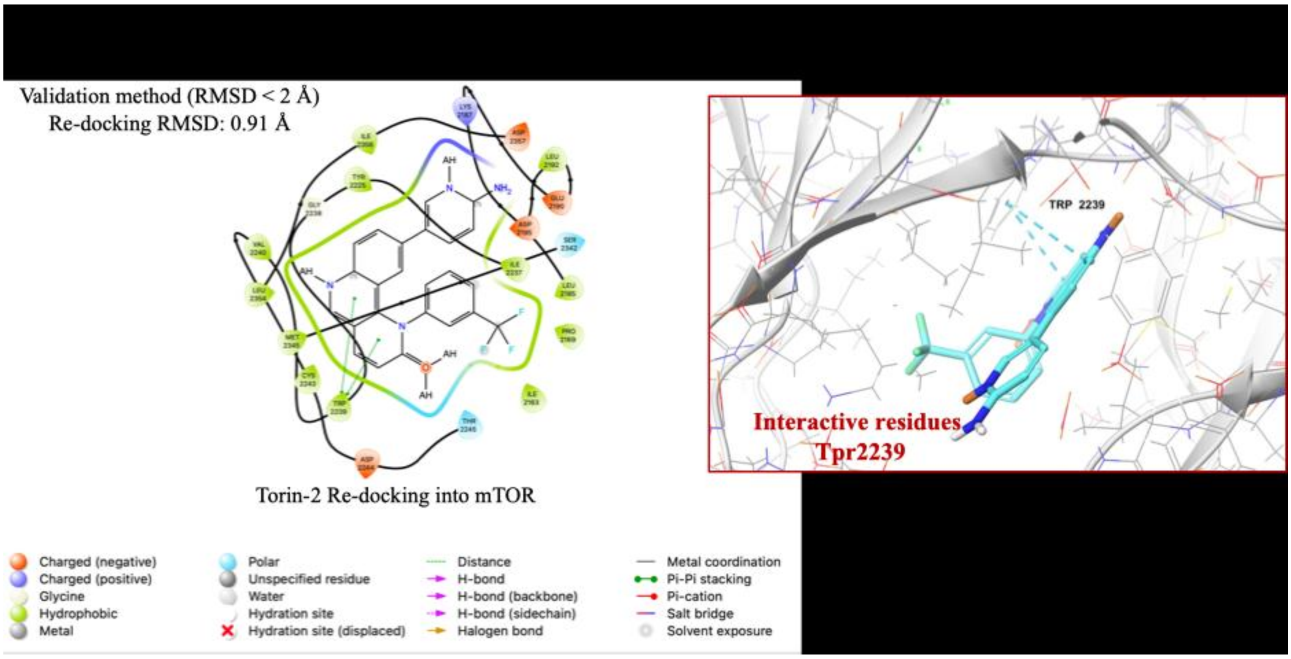

Figure S3. Validation by re-docking between Vioxx and COX-2

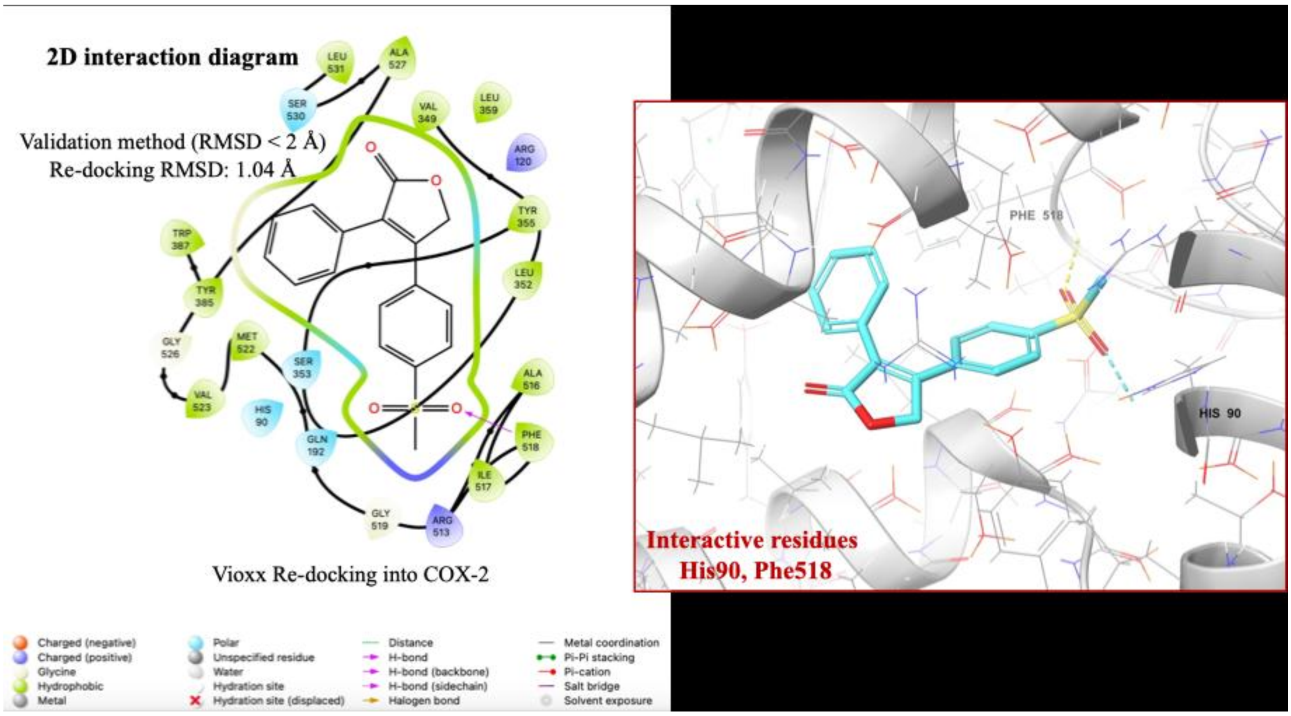

Figure S4. Validation by re-docking between BCL201 and BCL-2

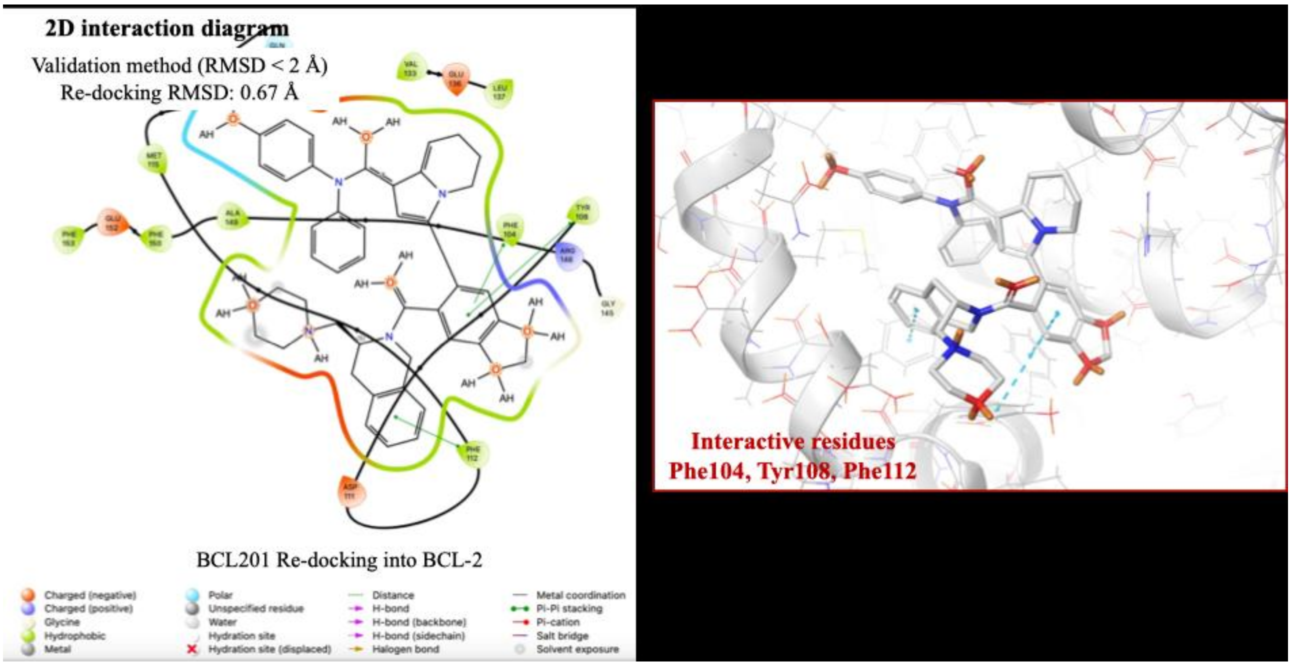
2D interaction diagram
Validation method (RMSD < 2 Å)
Re-docking RMSD: 0.67 Å
BCL201 Re-docking into BCL-2
Interactive residues
Phe104, Tyr108, Phe112
